# Parent and early childhood educator perspectives of unstructured nature play for young children: A qualitative descriptive study

**DOI:** 10.1371/journal.pone.0286468

**Published:** 2023-06-07

**Authors:** Kylie A. Dankiw, Saravana Kumar, Katherine L. Baldock, Margarita D. Tsiros

**Affiliations:** 1 Allied Health and Human Performance, University of South Australia, Adelaide, South Australia, Australia; 2 Innovation, Implementation and Clinical Translation in Health, University of South Australia, Adelaide, South Australia, Australia; 3 Alliance for Research in Exercise, Nutrition, and Activity, University of South Australia, Adelaide, South Australia, Australia; 4 Teaching innovation Unit, University of South Australia, Adelaide, South Australia, Australia; UBC: The University of British Columbia, CANADA

## Abstract

Nature play is growing in popularity, with many early childhood settings transforming their outdoor play environments to incorporate more natural elements. Current research highlights the benefits of engaging in unstructured nature play for children’s health and development; yet little is known about the experiences of key nature play end-users such as parents and early childhood educators, even though they directly impact the application of nature play within early childhood settings. This study aimed to address this knowledge gap by exploring parent and early childhood educator (ECE) perspectives to gain an understanding about their experiences with nature play. Using a qualitative descriptive approach, semi-structured in-person and telephone interviews were conducted with 18 ECE and 13 parents across four early childhood centres (from various socio-economic regions) across metropolitan Adelaide, South Australia during 2019–2020. Interviews were audio-recorded and transcribed verbatim. Thematic analysis identified five main themes; positive affirmations of nature play, factors influencing nature play engagement, defining nature play, outdoor play space design and risky play. Children’s connection to the natural world, learning about sustainability, emotional regulation, and children discovering their own capabilities were perceived advantages of engaging in nature play. Despite the benefits, ECE’s described institutional barriers such as resourcing, adhering to policies and scheduling conflicts, whereas, parents described time, getting dirty and proximity to nature play spaces as barriers to nature play engagement. Parents and ECEs alike described adults as gatekeepers for play, especially when other daily tasks compete for their time, or when faced with weather-imposed barriers (cold, rain, extreme heat in summer). The findings suggest that parents and ECEs may need additional resources and guidance on how to engage with nature play and how to overcome barriers within early childhood settings and the home environment.

## Introduction

Outdoor play spaces for children have been rapidly transforming in recent times [1], from traditional manufactured playgrounds into more nature-based play spaces with a focus on incorporating natural elements and features [2, 3]. The popularity of nature play may be attributed to emerging research that suggests nature play provides children with numerous health and developmental benefits, such as, emotional, physical, learning, and cognitive outcomes [4–6]. The recent evidence linking nature play to health and developmental benefits suggests it may be an important initiative to help promote children’s health [7].

Nature play is a type of outdoor play, that occurs *“in a natural environment and/or involves interaction with natural elements and features (e*.*g*., *water and mud*, *rocks*, *hills*, *forests*, *and natural loose parts*, *such as sticks*, *pinecones*, *leaves*, *and grass)*” [8]. Importantly, the overarching definition of play still applies in that play itself is a rewarding and fun activity that is usually (but not always) intrinsically driven/self-directed [8]. Nature play spaces are being implemented within schools, recreational park setting and within early childhood settings, as research suggests that children prefer nature play compared to traditional playgrounds [9–12]. Despite children’s preferences for nature play, opportunities for young children to engage in nature play may be influenced by other people in their lives [13, 14].

Many Australian children are spending a large amount of their time in formal care facilities, such as childcare or pre-school services. In 2011, children aged 2–4 years spent 22 hours per week on average in formal care facilities, with 52% in care 3–5 days per week [15, 16]. As children are spending more time in formal care, outdoor play spaces in early childhood settings may provide an important opportunity to promote play and facilitate health outcomes. Moreover, ECEs may play an important role in enabling children to access outdoor play spaces to meet their needs and facilitate engagement [17–19]. The literature suggests that parents and ECEs may support the shift towards more nature-based play [18, 20].

For instance, Herrington (2008) used focus groups to explore ECE perspectives at 14 centres in British Columbia regarding outdoor play spaces for children, and found 11 positive responses to incorporating plants in the children’s outdoor environment compared to four responses for no plants [18]. In addition to Herrington’s findings, more recent studies have found that parents and ECEs may face challenges when engaging in nature play. A study conducted by Tink and colleagues explored Canadian ECEs, primary school teachers and other service educator perspectives of nature play through interviews and found that other play activities were preferred over nature play [21]. Parents and ECEs perceived nature play as a risky activity with fears of child injuries, and liability being a main deterrent to engaging in nature play [21]. Little and colleagues [22] noted similar findings during semi-structured interviews with mothers of children aged 4–5 years regarding their perspectives of risk taking in outdoor play. Little and colleagues [22] found that while mothers emphasised the benefits of risky unstructured play outside, tensions existed between the desire to provide such play opportunities and fears about child safety. This highlights the complexities underpinning the decision-making process for parents and ECEs when weighing up potential risks versus benefits of nature play engagement.

Whilst Little et al. [22] and other research in different geographical contexts [18, 21] have identified and explored possible factors that influence unstructured nature play uptake, little is known about how parents and ECEs experience and perceive nature play in early childhood settings within a South Australia context. Therefore, this study addressed these knowledge gaps by exploring parents and ECEs perspectives of nature play adopting a qualitative descriptive methodology to gain a rich and detailed understanding about nature play experiences within an early childhood setting.

## Methods

### Study design

A Qualitative Descriptive (QD) methodology was used to explore nature play perspectives of parents and ECEs. The QD methodology was ideally suited as it involves obtaining rich descriptive experiences from participants to explain processes behind unique phenomena, which aligns with the aims of the research [23–25]. Similar research in the field of nature-based play and learning has utilised qualitative descriptive methodology, such as Miller and colleagues [3] which investigated the perspectives of teachers and principals regarding nature based learning in Australian primary schools. Neergaard and colleagues [23] argued that even though QD may not be as theory-based when compared to other qualitative methodologies, it is favoured when little is known about a phenomenon. No theoretical strings are attached to QD methodology; therefore, the analysis of the data stays true to the participants’ points of view [23]. In practice this was achieved by employing rigour strategies during data collection and data analysis (discussed further on), which include credibility (results are believable), dependability (accounting for the changing research context), transferability (results can be reproduced to other contexts or settings) and confirmability (degree to which the results could be confirmed by others) [24, 26]. This research was informed by, and reported using the COREQ [27] (consolidating criteria for reporting qualitative research) qualitative reporting checklist (see S1 Appendix).

### Participants and settings

The target sample were parents of children aged 3–5 years and early childhood educators (ECEs). Participants were recruited in January 2019 –June 2019 from four distinct early childhood centres (Centre A, Centre C, Centre CG, Centre S) located in Adelaide, Australia. Purposive sampling was employed to select both participant groups and the study sites. Purposive sampling selects participants based on individuals that have a story to tell, where the focus is on the phenomena of interest rather than aiming to offer a representative sample, or to draw inferences to the population (which is not the focus of qualitative research) [28]. Purposive sampling allows for the investigation of divergent participant perspectives [29], particularly when not much is known about the phenomena of interest (nature play). Sample size in qualitative research can be difficult to estimate as there are no definitive guidelines [30]. For the present study the sample size was influenced by several considerations. Firstly, by reviewing similar studies that have used qualitative descriptive methodology, such as Miller and colleagues [3] who interviewed teachers about their perspectives of nature based-learning. Secondly, informed by recommendations from prominent qualitative methodologists such as, Creswell [24], Sandowloski [31] and Neergaard [23]. Thirdly, continuing to collect data until such time as similar findings were repeated by participants (saturation) [32]. Finally, based on time and resource constraints as the lead author was a higher degree research candidate who had to adhere to project timelines. All parents with children aged 3–5 years attending one of the centres, and ECEs employed at the same centres, were considered for inclusion. Four privately operated early childhood centres were selected for the study based on their sociodemographic location and willingness to participate in the study. The four early childhood centres were privately managed centres located within diverse socio-economic zones, referred to in Australia as Index of Relative Socio-Economic Decile Ranks (SEIFA). The decile ranks were obtained from the Australian Bureau of Statistics Socio-Economic Indexes for Areas five-year census data [33]. The census index data is presented per state and suburb area by describing different aspects of socio-economic advantage and disadvantage. The lowest (more disadvantaged) 10% of areas are given a decile number of 1, the next lowest 10% of areas are given a decile number 2 and so on, up to the highest (less disadvantaged) 10% of areas which are given a decile number of 10 [33].

### Recruitment

Early childhood centre directors/ECEs disseminated information packs (containing a flyer, information sheet, consent form and return envelope) to parents of all children who attended the centre via email/hard copy. A member of the research team was also on site to provide clarification, or a replacement information pack. Interested participants returned a signed consent form to the front desk at the centre if they wished to participate. Recruitment of ECEs involved a similar process.

### Data collection

One-off semi-structured interviews were undertaken with consenting parents and ECEs. Semi-structured interviews were most suitable compared to structured interviews as the former allows the researcher to have open conversations with participants, using open-ended questions that are free-flowing, allowing the participant to elaborate more detail and clarify matters [34]. Before the interview, participants were advised that their responses would be recorded using a handheld audio-recorder. All interviews were conducted from July 2019 –January 2020. One female post-graduate research student (KD) conducted all interviews. All parent interviews occurred over the phone (n = 13) whereas ECE interviews occurred over the phone (n = 9) and face to face at the centre in a separate space where only the participant and the student (KD) were present (n = 9). Parents were not known to KD. However, as KD had visited each of the centres several times, some ECEs were familiar with the student. Interviews were conducted using an interview guide which included questions and prompts for participants (see S2 Appendix). The interview guide was developed through consultation with the supervisory team and informed by a review of the literature based on qualitative studies which explored parents and ECE perspectives on play for young children [35–37]. The guide was used as a tool to help the interviewer and was not given to the participants. The interview questions were centred around the use of nature play for young children, in regard to participant experiences, beliefs, perspectives, description of nature play and the barriers and enablers to nature play engagement. Demographic information collected from participants during the interview included gender, occupation, postcode [later converted to decile rank (SEIFA)], highest education level, and where participants grew up and lived as young children. Interviews lasted 20–40 minutes and were audiotaped and transcribed verbatim (online transcription company Rev; https://www.rev.com/) and no field notes were taken. It is important in qualitative research to acknowledge and reflect on the possible assumptions, beliefs and experiences of the research team, which may influence data collection and analysis, referred to as reflexivity [26]. The post graduate research student’s past experiences with nature play were first established during her Bachelor of Science degree (environmental science and biology) and influenced her chosen masters and doctoral project. The student and research team acknowledged their personal beliefs about nature play, and collectively each member described themselves as an advocate for nature play and these beliefs were regularly discussed and documented in weekly meetings during data collection and analysis. The student had knowledge and training on qualitative principles prior to conducting the interviews through workshops held by the University of South Australia. Prior to interviews being conducted, the candidate underwent training with the supervisory team to practice interview techniques by conducting mock interviews and pilot testing the interview guide. Feedback was provided by the supervisory team. One member of the research team (SK) in particular, is an experienced qualitative researcher and provided the candidate with expert mentorship during the data collection and analysis phases of the research.

### Analysis

The data from the semi-structured interviews were managed using NVivo software (Australian version 10). Thematic analysis was used to analyse, identify and report patterns (themes) that emerged in the data to give a rich description of the phenomenon [38, 39]. The six phases of thematic analysis undertaken included: familiarising one’s self with the data (reading data); generating initial codes (systematic and relevant coding of data); searching for themes (organising codes into themes,); reviewing themes (checking if the themes are appropriate in relation to the coded extracts); defining and naming themes (analysis of how themes are defined); and finally, producing the report [38]. Biases and assumptions were managed during the analysis by firstly taking an inductive approach to interpreting the data, meaning that the research team sought information out from the interview data to create themes, rather than taking a deductive approach. Inductive reasoning looks for meaning from within the participants rather than from the perspective of the researchers interests or self-fulfilling intent [40].

Rigour strategies were employed to ensure the results were credible, dependable, transferable, and confirmable during the analysis process [26]. Credibility was maintained by employing investigator triangulation, whereby all members of the supervisory team participated in the coding and analysis process by reviewing each stage of the thematic analysis and providing feedback. Dependability was maintained by firstly using rich codes to support the development of the themes generated. Secondly, the use of direct quotes with thick, rich descriptions was sought out to further support the development of the themes. Thirdly, a diverse selection of participant codes were used to support themes developed; that is the codes attributed to the themes were drawn from multiple participants. Transferability was achieved by having regular meetings with the research team during the analysis phase. The meetings with the supervisory team were documented by the candidate to include topics of discussion and decisions made. Confirmability was maintained by regularly discussing and documenting the assumptions and biases identified by the supervisory team, with the aim of the meetings to assess the peer review process and contribute to peer coding of theme development. This involved each member of the research team peer reviewing an interview transcript for codes and themes independently, and once completed the team discussed the differences between the themes and codes generated from each member. Transcripts were not able to be returned to participants due to practical limitations.

### Ethics

Ethical approval to conduct this research was given by the University of South Australia Human Ethics Committee (HREC) (Protocol no. 201137). Participation was voluntary and informed, meaning that participants could withdraw at any time without any consequences and were given sufficient information and understanding about the study. Participation was confirmed once written consent was given by the participant.

## Results

**Fig 1:**
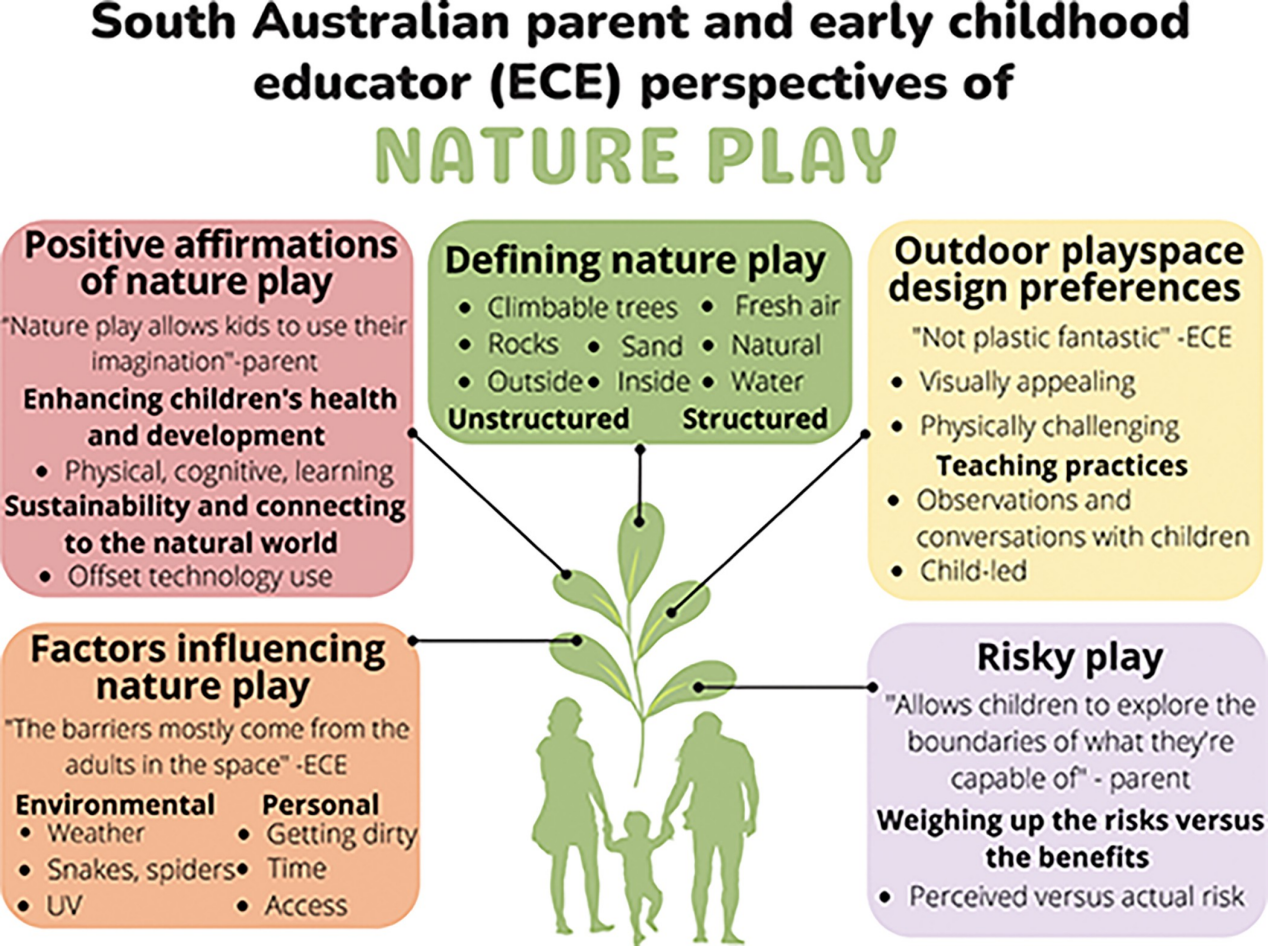
Diagrammatic overview of themes and sub-themes.

Recruitment yielded interest from 27 ECEs and 29 parents. Of these, 18 ECEs and 13 parents consented to take part in the semi-structured interviews. Of the ECEs who did not participate, 9 did not respond and 1 declined. Of the parents who did not participate, 15 did not respond and 1 declined. Thematic analysis of the interview data identified five main themes; (1) positive affirmations of nature play, (2) factors influencing nature play engagement, (3) defining nature play, (4) outdoor play space design preferences, and (5) risky play. Sub themes were also generated from each of the main themes. A diagrammatic overview of the themes and sub-themes is shown in Fig 1.

Table 1 provides an overview of the demographic characteristics of participants. Table 1 indicates that majority of the participants were female and born in Australia, with most parents completing an undergraduate degree compared to majority of ECE’s whom completed a diploma with 4–7 years’ experience as an ECE.

**Table 1:** Demographic characteristics of parents, Early Childhood Educators (ECE) and their centres.

	Parents	ECE
	n (%)	n (%)
**Gender**		
Male	2 (15)	2 (11)
Female	11 (85)	16 (89)
**Highest level of education**		
Certificate	0 (0)	4 (22)
Diploma	0 (0)	8 (44)
Undergraduate degree	7 (54)	5 (28)
Masters degree	5 (38)	1 (6)
Doctorate degree	1 (8)	0 (0)
**Educator experience**		
0–3 years	NA	3 (17)
4–7 years	NA	7 (39)
8–13 years	NA	3 (16)
14–17 years	NA	1 (6)
18-21years	NA	2 (11)
22+ years	NA	2 (11)
**Place of birth**		
Australia	8 (62)	13 (72)
China	0 (0)	1 (6)
Malaysia	0 (0)	1 (6)
England	3 (22)	0 (0)
Philippines	0 (0)	1 (6)
South Africa	0 (0)	1 (6)
Sri Lanka	0 (0)	1 (6)
Argentina	1 (8)	0 (0)
Poland	1 (8)	0 (0)
**Socio-economic Indexes for Areas in South Australia (SEIFA) rank**		
Centre A (rank 9)	5 (38)	5 (28)
Centre C (rank 5)	3 (22)	7 (39)
Centre CG (rank 2)	0 (0)	3 (17)
Centre S (rank 2)	5 (38)	3 (17)

*NA = not applicable

### Theme 1: positive affirmations of nature play

The first main theme identified distinct perceptions from both parents and ECEs which described nature play as being beneficial for children and was preferred over other play experiences (plastic toys, use of technology). One ECE described:

“I think, personally, that nature play is good for the children. It gets them all dirty and not playing with all the plastic stuff out there. I think that’s not as good as nature” (participant 1, centre A, ECE)

Similarly, parents and ECEs alike described that nature play can be used to offset increased technology use by fostering a connection to nature. Presented with these two concepts, two subthemes were identified: (1) enhancing children’s health and development and (2) sustainability and connecting to the natural world.

#### Subtheme 1: Enhancing children’s health and development

Parents and ECEs alike described nature play as being beneficial to children’s overall health and development, specifically, learning outcomes, developing a sense of self, physical, emotional, and cognitive development such as imagination and creativity. This subtheme is further emphasised by the below extracts:

“Nature play allows kids to use their imagination to go a bit wilder. They can invent. Take wooden trunks and trees and, turn them into things” (participant 2, centre C, parent)

“Whereas with Nature Play they can go and climb a tree, or hide in some bushes or something then that, they get to choose that, so there’s that sense of agency, as well, that they get from choosing their play which then leads to choosing their own learning and their own learning path” (participant 1, centre S, ECE).

#### Subtheme 2: Sustainability and connecting to the natural world

Parents and educators alike described how nature play can be used to offset technology use, such as TV and screens, resulting in children forming a connection to the natural world where they are able to learn about sustainable practices. This concept was emphasised by both parents and ECEs:

“Interacting with nature helps teach them about sustainability and their place in the community and the world and part of who they… like the being, belonging, becoming which is one of our practices in the early years learning framework. It really helps sort of solidify that element in a child’s life” (participant 7, centre CG, ECE)

“Coming out with nature play, I think it’s probably a bit of a response to screen time and the detriment that its actually causing and we’re seeing a lot of our kids are addicted to screens” (participant 1, centre A, parent)

### Theme 2: Factors influencing nature play engagement

Whilst nature was perceived in a positive position for children, both parents and educators described their struggles in engaging children with nature play in terms of personal and environmental factors. Adults were also described as a barrier in terms of the inherent control they have over gatekeeping where children play. Educators may limit access to nature play when they are faced with institutional challenges such as following safety regulations, having to clean and maintain the play space and having enough time in the day to do so, particularly when nature play activities were described as being messy and dirty by both parents and ECEs. The extracts below highlight these struggles for ECEs:

“The educators are probably a bit of a barrier sometimes. Like for me if I ever got the hose out, I’ll spray the kids because I know they love it. But I know some are like, "Ah", that then means that they have to change the child. So, I think sometimes educators can be a boundary, for sure. You know, not wanting to put mud in the mud kitchen because then the child gets dirty” (participant 4, centre S, ECE)

Communicating to parents that children may get messy and dirty during play time is a challenge for some educators. In privately operated centres, educators described that the parent is essentially a customer, so they are restricted to how they deliver nature play content to children when some parents do not support the messy and dirty aspect of it:

“And parents can get a bit funny as well. Even with, like water play and stuff like that. Some families are, you know, "Why are they wet?" "Why are they dirty?" Right, so you have to really… choose your times” (participant 1, centre C, ECE)

Children don’t want to get dirty and disappoint their parents. When they come up and the first thing they say, "Oh, my goodness. Look how dirty you are. Cause I do know, I hear so many comments like, "Ah, I have to be clean. Mommy will get cross, or daddy will get cross." (participant 5, centre CG, ECE)

Parents further described time as being a factor, whereby nature play activities may be constrained to make room for other more important tasks in the day, such as work and family life. For some parent’s nature play spaces are not readily accessible, and may take added time to get to, highlighted by the below extracts:

“I think other barriers are time because I feel like a lot of Nature Play isn’t readily accessible it’s just on the outskirts of Adelaide, it’s that little bit further out and I’m a shift worker, so a lot of the time we don’t often go to those places” (participant 1, centre C, parent)

#### Sub-theme 1: Personal factors

There was a focus on participants describing personal and professional experiences with nature play and how these experiences shape their approach to nature play for children. Some ECEs described that they have attended nature play workshops which provided them with ideas on how they can engage children with nature play:

“My view massively changed when I started working in a service. They’re connecting with the natural environment and obviously we’ve been to a few like, PD days where we’ve talked about nature play, why it’s important and stuff and I think that and I’m a very creative person. I love getting messy and you know, obviously I don’t mind the child getting messy. I think it’s fantastic. I love that” (participant 4, centre S, ECE)

Parents and ECEs emphasised that their past childhood play experiences with nature play influenced the types of nature play opportunities they give to children, whereby they are wanting to provide children with similar play experiences to their own:

**“I think also, what I mentioned before, because I grew up in the** countryside and those memories for me are the best in my life. And everything with nature play, I remember all those things. I really value those memories. So that’s what I want for my child” (participant 1, centre S, parent)

One parent described that even though they were not raised in a nature-oriented environment and experienced nature play periodically, their attitudes towards nature play were positive, and now they provide nature play experiences to their own children:

“I was raised in communist Poland in a one-bedroom apartment and my nature play consisted of a sandpit in between the massive apartment blocks, which was one of the only pieces of equipment for all the village children out there. I just crave nature. I love camping, I love being outdoors and I probably just would have done that for my kids because I enjoy it and I see the benefits that I get from being outside” (participant 2, centre S, parent)

Many participants described that like ECEs, they found the dirty and messy aspect of nature play conflicting. One parent described how they struggle with the importance of mud play against children getting muddy:

“I don’t particularly enjoy it when they get super muddy but I try to just roll with that because I think it is really important for them so mentally I have a bit of an inner struggle I guess. That could be just for me but I try to just over-rule that” (participant 5, centre A, parent)

One parent who is also a primary school teacher, described how frustrating it is to transition the children back into the classroom after they have been outdoors collecting loose parts and tracking them back inside:

“Well, the kids get very dirty, logistically. We spend a lot of time washing their feet and putting their socks and shoes back on. I initially thought, oh my goodness, they can’t possibly take their shoes and socks off outside. But then that was part of the experience for them and digging in the sand with their feet. So that was quite frustrating for me as a teacher. And we’re trying to transition all activity to classroom we just want them to be clean. Also, some of the heavy lifting and the, sticks that they come up with, they tend to just, try and put them in their bag and take them home, so those kind of things” (participant 5, centre S, ECE)

Parents described how personal circumstances may influence how they engage with nature play, such as proximity to nature play spaces, competing child interests and having enough energy to provide those nature play experiences for their children:

“We have an interesting situation at home, where I’m not a single parent but my husband is sick and works nights. So, a lot of the time, it’s just me with my child at the moment, I’m pregnant with our second child. So, I feel like I don’t get to have those nature play experiences as much as I would like to because I’m kind of, trying to get through the day on my own with her. Four-year-old’s are hard work” (participant 1, centre A, parent)

Participants described how personal and individual child needs are also a factor, such as making sure nature play spaces cater for different age groups and ensuring they are safe:

“Obviously, if you’re having shared spaces for the, babies, toddlers, and then you’ve got the older age groups sharing the play space with the babies, things like rocks don’t really work in that environment, because they’re picking it up, putting it in their mouth. Which is hard because especially if they’re sharing the same play space, you got to cater for babies, plus toddlers, and then putting an environment to cater for both” (participant 10, centre C, ECE)

Several participants described that some children may need more guidance than others when it comes to engaging in nature play activities. Not all children may enjoy getting wet or dirty and may need more encouragement, whilst other children’s enthusiasm may require more supervision, as emphasised by parents and ECEs alike:

“I think that for my daughter… Because she’s very energetic, she needs some guidance and sometimes she entertains a lot. And with water play, she is watering the plants a lot. So, that needs a lot of guidance, of course” (participant 1, centre S, parent)

“Obviously some children need more guidance with their play than others and need a bit more initiative I think, sometimes though children feel a bit lost if they don’t have that kind of guidance and that thing to focus on, especially those that struggle to initiate play. I sometimes see children wandering around and looking for ways to engage which is, I guess, is where we come in and try and guide them to choose something that they like” (participant 1, centre C, ECE)

#### Sub-theme 2: Environmental factors

Weather and temperature were found to be factors impacting nature play engagement for both parents and ECEs, particularly in Australia as participants described the high temperatures in summer as a barrier to going outside, along with rain and cold weather in the cooler months, such as the following:

“So I would say the weather is a very, very big contributor. When it comes down to it, if it’s really cold and miserable and raining, I am not one to be outside. And if it’s 40 degrees, I don’t want to be outside either. So, other than that I’m pretty happy. If the weather is good, I’m good” (participant 1, centre A, parent)

“Just the weather is a very hard one. You know, like when it’s freezing cold we will go out there, but just for a quick run around and then come in. Same with the hot weather. So I feel like the weather is just our barrier really” (participant 4, centre A, ECE)

Whilst parents and ECEs identified weather and temperature as influencing factors, they both recognised that they may be overcome by making sure children have appropriate wet weather gear (rain jackets, gum boots), having appropriates shade and bringing the outdoors inside, as one educator described:

“The girls in the baby room were like, "Oh, it’s hot. What do we do?" So, I got them to bring in a sand pit yesterday into the baby room” (participant 6, centre CG, ECE)

Some educators had concerns about bringing nature play activities inside, describing that it may be costly, require resourcing or may damage the indoor area, emphasised by the below extracts.

“In that regard, it’d be nice to have like some resources that we can use inside the room, so you could have, little pebbles and ponds” (participant 7, centre CG, ECE)

“And money, as well. I know it is easy to grab everything that’s outside and bring it in, but I guess you have to think about, you know, is the sand going to scratch the floor?” (participant 4, centre A, ECE)

### Theme 3: Defining nature play

Participants were asked about what nature play means to them, and they described nature play as being outdoors, both structured (purpose built) and unstructured (naturally occurring) and child-led where children are able to explore their surrounding within a natural setting. Participants detailed nature play activities as climbing trees, building cubby houses, and making mud pies in environments such as creeks, national parks, coastal environments, along with having available natural materials to engage with such as bark, sticks, rocks, leaves, and living things such as insects and animals:

“there’s so many different types of nature play I guess. (laughs) Generally we think of the mud, the dirt, the water, that kind of thing” (participant 1, centre S, ECE)

“So, nature play to me, just means being out, the kids being out in nature and engaging in natural environments, so you can climb trees or you can gather some leaves, or play with mud. So that, that’s what I’m thinking” (participant 1, centre A, parent)

Interestingly some ECEs described that nature play spaces can be mixed play spaces, with aspects of artificial features balanced with natural features as one educator described:

"What other aspects do you have, if you only have concrete, soft fall, and artificial lawn, then no. But if you have artificial lawn, but then you have bark, and you have sand, and you have dirt, then you have a balance” (participant 2, centre S, ECE)

Whereas some participants simply described nature play as being outside, with no plastic features.

“Mostly I really think it’s just about being outside, for me” (participant 5, centre A, parent)

“To me it looks like nothing plastic” (participant 4, centre A, ECE)

Compared to several others who believed nature play can be indoors as well.

“Oh, everything outdoors. And even in indoors I’d like to incorporate nature play indoors as well” (participant 9, centre C, ECE)

Whilst participants alike spoke of similar features and elements that make up nature play environments, confusion around what constitutes a nature play space was shared by both parents and ECEs, as described below:

“So, whether we’re doing it correct or not, whether we’re going on the right path, that’s a bit of a confusion for me. So, I would say my knowledge about nature play is not great. I want to learn more” (participant 2, centre C, ECE)

“But I think, I wouldn’t know where to go to find parks that have any kind of nature play in them. It’s like you’re trying to sell something, but people don’t know what you’re selling” (participant 5, centre S, parent)

#### Sub theme 1: Nature play can be structured and unstructured

When participants were describing what nature play means to them, they were also asked about structured (purpose built) and unstructured (naturally occurring) nature play, in terms of what it looked like and how they define each of the contexts. Parents and ECEs perceived structured nature play spaces to be safer than unstructured nature play spaces due to safety concerns surrounding the unknown dangers that may exist in a naturally occurring spaces. Some of these concerns were described as risks unique to the Australian bush context such as, spiders or snakes, compared to early childhood settings where the equipment is regulated, and nature areas are maintained:

“I guess if it’s structured like our centre, it would potentially be safer. So, if it’s out in the bush, you don’t know what’s out there. There could be more hazardous items which, obviously, you can’t control that. Like being out in the bush if they don’t have shoes on there could be a snake they might step on or if it’s in a childcare centre, if their shoes are off, there shouldn’t be anything too dangerous for them, to hurt themselves with” (participant 1, centre A, ECE)

“How do I explain clearly? The… childcare centre, the environment’s pretty reasonably controlled, there are regulations around play equipment” (participant 3, centre S, parent)

One parent described their experiences and perceptions of a purpose build nature play space they have been to:

“What this National Park gives, for me, is I get some sort of an implicit reassurance that it is safe and has been assessed. Even though it’s made up of natural things like logs and things, I think that because it’s, it’s set up as a park and it’s, been designed by somebody, for me, that comes with an overarching sense of security around that this is actually appropriate for kids to play on” (participant 2, centre C, parent)

One educator explained that dangerous hazards can also be found in a structured nature play space within early childhood settings, emphasised by the following.

“Some parents freak out. Like, "There’s a spider out in the yard, and you need to go get the fly spray." I said, well, where do spiders live? They live outside." "It’s not harming anyone. Leave the spider" (participant 6, centre CG, ECE)

### Theme 4: Outdoor play space design preferences

Participants were asked about what features and elements they would like to see in a nature play space. Participants often referenced their own current centre and spoke about play areas they liked and disliked, along with improvements they would like to see such as less plastic and more real nature elements like real grass:

“I’d like to see the whole back garden, the actual plants, where we’ve got the vegetables and stuff growing, I’d like to see that sort of improved a bit more so we can get more children in there, get more children involved. I mean in a dreamworld, we’d have real grass” (participant 7, centre CG, ECE)

“I feel like I would want grass. Dirt obviously to play in and I would love if we had, at our centre we’ve got a lot of climbing equipment, but it is all plastic. It is man-made, and it’s not as natural” (participant 4, centre A, ECE)

For some parents it was important for the nature play space to be visually and aesthetically appealing, as highlighted below:

“some of these companies are pretty spectacular looking, they’re very artistic in the way they present, and they’re not just a bunch of wood stacked on top of each other, there’s real sort of design concept behind them in terms of aesthetics, and I think that really is actually going to help people get in to it” (participant 6, centre A, parent)

Whereas for some ECEs they would like to see more physically challenging structures:

“I think our yard needs a bit of an upgrade in terms of physical adaptions so that the kids have a bit more of a challenge and doing that in a natural way. I think more like, physical climbing stuff. Some more climbing equipment but in a natural way” (participant 1, centre C, ECE)

“More like obstacle course kind of resources, so we have a lot of wooden planks and tires and tree stumps, but I’d like to see more like larger rocks maybe, so- something that’s physically heavy for the children to lift” (participant 7, centre CG, ECE)

#### Sub theme 1: Teaching practices

The ECEs consistently spoke about the importance of considering children’s own preferences for play when designing nature play spaces or creating activities. Many ECEs described this as child-led play, whereby the programming of activities they deliver is based on conversations and observations they have had with the children:

“We do program for activities, but I think a lot of is all stemmed from the child’s interest and the children love being outdoors, they love being able to engage with the natural environment, and so a lot of the programmed activities are therefore based around that” (participant 7, centre CG, ECE)

Some ECEs identified that they do highly influence where the children play, but by asking children open ended questions they are able to guide their play without controlling it, as described by one educator outlining how they scaffold children’s learning:

“I’m always trying to scaffold their learning, as well. I like talking about what they’re doing and trying to expand on what they’re doing” (participant 2, centre A, ECE)

### Theme 5: Risky play

Participants were asked about what risky play means to them, such as their experiences and likes and dislikes. Questions about risky play were explored further due to how parents and ECEs consistently described nature play as having a risky element or children getting out of their comfort zone, such as climbing trees, playing in bodies of water, and playing with sticks and rocks:

"Risky play means kids being able to take risks that they feel comfortable with, to improve their understanding of the physicals of the world, really. So, you know climbing a tree is a risky play” (participant 1, centre A, parent)

“Just opportunities for them to feel a bit more out of their comfort zone. We did a lot of tree climbing and stuff like that to make them feel more confident to approach those situations.” (participant 1, centre C, ECE)”

#### Sub theme 1: Weighing up the risks versus the benefits

Parents and ECEs alike identified that children engaging in risky play was beneficial, in terms of being a learning opportunity for children. Children can learn about their own physical capabilities, their environment, abilities, boundaries, and build confidence whilst having a sense of agency over what they are comfortable with, essentially setting their own limits for play as parents and ECEs alike described:

“Risk play, when I hear that word I think of it being something really good actually, because risk play is so important for children. It’s so important for them to learn their boundaries, their limits, and to challenge themselves. I highly, highly recommend risk play. I really think it’s good for children (participant 4, centre A, ECE)

However, tensions exist for both parents and ECEs between weighing up the benefits of risky play against the perceived risks, as highlighted below:

"Well I saw the pump being used at school and I thought, Oh my gosh, someone’s going to get their fingers trapped or, should they be jumping on those tyres? And everyone said, No it’s okay, they’re allowed to explore and feel their own environment, rather than have it all, sort of, pre-made for them” (participant 5, centre S, parent)

Some parents described that they themselves were risk adverse and thus do not encourage risky play with their children, as highlighted below:

“We’re all not risk takers, we don’t (laughs) engage in risky play too much, I don’t like it” (participant 8, Centre A, parent)

One parent described themselves as a non-adventurous and a ‘helicopter parent’, and emphasised that the risky play opportunities ECEs provide are crucial, particularly when they are not able to provide them at home:

“I’m the helicopter parent. It’s, her dad saying "Let her go and be adventurous." Type parent. So when I’m there, I am very much the, "Hang on. Don’t fall down. Be careful. What are you doing Blah, blah, blah." So, in that sense, it’s good that this does happen at childcare (laughs) when I’m not there because it allows them to do what I know in my head is really important. But when I’m there I do get concerned that they might hurt themselves” (participant 2, centre C, parent)

The ECEs whom want to provide risky play opportunities at their centre may be restricted to do so due to rules and laws about equipment use and safety concerns, as one ECE described:

“I just give them the opportunity to plan and to jump. In our place the retaining wall is not even too high, but they are not allowed to climb up on any of these retaining walls. And if they will jump off there, and one child has done so, it’s impossible for him to get hurt and I’m restricted there. There’s nothing I can do. I tried that but they just said, "It’s a law and we have to keep the children safe” (participant 5, centre A, ECE)

One ECE suggests a way to ease these tensions is to educate parents about the real versus perceived risks and to communicate the benefits on how to alleviate real risks instead of removing them completely:

“So, I think that probably education around the real versus the perceived risk, is probably the only way to, to get over that because I actually don’t think you can actually change or remove those risks because then you’re not achieving what I think are some of the greatest beneficial outcomes from nature play, if you start changing what the kids are actually exposed to. So, it’s probably about that, educating the parents, I think” (participant 2, centre C, parent)

Educators and parents agreed that the risks provided to children should be age appropriate and the individual abilities and capabilities of children should be sought out before encouraging risky play, as one ECE described that knowing the abilities of individual children is important when encouraging risky play:

“So we just have to let them take the risk, but that depends on the child and the families obviously and the age or the development of the child” (participant 2, centre C, ECE)

Similarly, a parent described how she factors in safety and the age of her child before engaging in risky play:

“So, so, I certainly do factor in her safety, what I think is age appropriate for her and I probably do err on the side of protectiveness” (participant 2, centre C, parent)

## Discussion

Research investigating the importance of nature play for young children has been steadily increasing [4], as countries including the USA [41, 42], Norway [11, 43], the United Kingdom [44], Canada [7], Scotland [45] and Australia [2, 44] are reporting on the numerous health and developmental benefits associated with the nature based re-design of children’s paly spaces. Despite the increasing uptake of nature play in early childhood settings [1], it is unclear what influences the application of nature play from the perspectives of nature play stakeholders, such as parents and ECEs. Parents and ECEs are important facilitators of nature play, and the qualitative descriptive methods utilised in this study allowed for in-depth exploration of their valuable perspectives. The contribution of this research is novel, as it is the first study to explore the perspectives of unstructured nature play for parents and ECEs within a South Australian context, with a focus on young children in early childhood settings. In contrast, previous research such as Miller and colleagues [3], has focused on the perspectives of parents/educators of primary-school children or out of school hours care (OSHC) [21]. Focussing on different ages, stakeholders and contexts adds to the literature in several ways. First, it contributes new knowledge in terms of how children of different ages engage with unstructured nature play. This is critical as it may be useful in informing strategies to promote nature play for different age groups. Second, exploring and understanding different stakeholders’ perspectives about nature play may assist in informing policies and practices which can promote enabling factors and mitigate challenges to nature play engagement. Finally, given the diversity of contexts and environments in which nature play can occur, it is important to understand how and what role contexts play in promoting nature play.

The findings of the present study provide insight into the complexities underpinning how parents and ECEs navigate nature play engagement within early childhood settings, which was closely linked to their perceptions and experiences with nature play. Parents and ECEs described that they encouraged nature play engagement because they believed it to be good for children, thus having positive affirmations about nature play influenced their decisions to engage with it. In line with current research [4], parents and ECE’s consistently described their view that nature play was important for young children’s health and development in terms of the physical, social, learning, emotional, and cognitive benefits. Notably, parents and ECEs described that nature play helps support children’s emotional regulation, noting that children were a lot calmer and happier after playing in nature, which is consistent with current evidence [4, 5, 46].

In addition to health and developmental benefits described by parents and ECEs, they also identified how engaging in nature play activities can foster children’s connection to the natural world, which may result in children developing positive attitudes towards sustainable practices in the future. A recent systematic review conducted by Ernst and colleagues [47] which consolidated the findings from 32 quantitative and qualitative studies found, children aged 3–5 years who participated in a nature play program at their early childhood setting improved their knowledge about plants, wildlife, living things, and established compassionate care for nature, as measured by observations, qualitative interviews, and questionnaires [47]. The findings from Ernst and colleagues [47] align with the findings from this study and taken together highlight the importance of nature play as a valid contributor to sustainability outcomes for children and the natural environment [47].

Parents and ECEs described how the bespoke natural environment that nature play settings provide may be a haven away from the emerging influence of technology and screen use, as parents described that nature play could be promoted as a way of offsetting technology and screen time for young children. Increased sedentary screen time use has been linked to a higher risk of obesity and mental health issues for young children [48], as time spent on screens may replace time spent engaging in activities that have been shown to benefit children’s health and development, such as PA [49] and outdoor play [50, 51]. Investing in strategies aimed at increasing PA and outdoor play, such as nature play, may act to inhibit screen time use for young children. Thus, future research examining the effectiveness of nature play to offset screen use may be warranted.

Defining nature play was identified as a main theme in influencing parents and ECEs delivery of nature play, as participants consistently described being confused about what nature play is. This notion is consistent with current evidence, as semi-structured interviews conducted by Miller and colleagues [3] also found that educator knowledge about nature-based learning were key barriers for 12 South Australian primary school teachers and principals. The lack of consensus on what constitutes nature play in the literature may have contributed to this knowledge gap. However, since the recent works by Lee and colleagues in 2022 have addressed consensus on how nature based-learning, nature play and other play definitions are defined, confusion for educators may be alleviated by presenting them with these findings [8]. Whilst nature-based learning is a structured activity that takes place in an educational setting, there were similarities between primary school teachers and ECE perspectives, along with sector/context-based differences. These similarities may be attributed to the lack of knowledge about nature play, which was shared by parents and ECEs alike, similar to the findings of Miller and colleagues [3]. For some parents and ECEs, nature play was simply defined as being outdoors, whilst some described it as needing to be within a natural environment with only natural materials, with others describing that nature play could be indoors or outdoors. It was evident that both parents and ECEs shared in their confusion about what nature play is and what its parameters are. Such uncertainty may not be a feature in the future, as recent research by Lee and colleagues outlines a consensus-based definition of nature play [8]. Leveraging from such research, it may be important to provide additional resources to parents and offer specialised training to ECEs about nature play definitions, principles and resources that can be adopted. Previous research by Busoni and colleagues [52, 53] conducted in Canada found that, providing parents and ECEs with additional training and resources, such as web based, and in-person workshops increases their tolerance in delivering and facilitating risky play. Whilst these findings are encouraging, future research may be able to adopt the methods utilised by Brussoni and colleagues [52, 53] to develop and evaluate specific nature play training and resources.

In addition to the ambiguity surrounding what constitutes a nature play space, ECEs mentioned that they need guidance on how to use natural resources and where to source them. Parents and ECEs also agreed that children may need extra support and guidance too, especially those who are shy and more reserved. Thus, it may not be enough to simply provide a natural environment and expect children to engage with it, there may need to be more thought around the affordances and the need for scaffolding of play experiences for some children. Play affordances have been explored by psychologist Gibson [54] and this theory has been established as an important framework to consider when designing play spaces and activities for children. In line with the descriptions made by parents and ECEs in this study, the theory is built on the notion that affordances are unique to the individual and unique to different objects and environments. For example, a tree may not afford climbing to an infant, but a tree with a straight, thick branch may afford an older child to climb on it [54, 55]. Whilst this notion was shared by parents and ECEs alike, that nature play spaces need to be age appropriate, a more viable approach would be to provide a variety of nature components with a nature play space so children of different ages can explore their own affordances and abilities.

Weather and temperature were identified as a main barrier to nature play engagement for both parents and ECEs. This barrier is also shared by other parents and ECEs within different geographical contexts and sectors. Miller and colleagues [3] explored perspectives of teachers and principals with a South Australian primary school context, which found that inclement weather can disrupt plans to engage in nature-based learning outside the classroom, such as extreme heat in summer and cold weather in winter. Similarly, Basbay and Atmaca [56] explored the perspectives of parents and educators in a Turkish forest school for children of primary school age and found that extreme snow conditions made it difficult to plan outdoor curricula [56]. Interestingly, a common practice in Finland is allowing young babies up to the age of 2 years old to sleep outside in the wintertime in temperatures ranging from minus 6 to minus 15 [57]. Parents who participated in this practice were interviewed and surveyed about their experiences and opinions, and the study found that parent’s experiences were mainly positive and most parents had not faced potentially dangerous situations [57]. One third of the parents surveyed agreed that this practice helped aided to toughen up their child, and prepare them for cooler conditions in the future [57]. Similarly, this study found that a small number of parents and ECEs agreed with this notion that being outdoors helps to toughen up children but did not reference harsh weather conditions as a way to achieve this. This may be due to the fact that the South Australian climate is very different to that of Finland, and in Australia the ultraviolet (UV) radiation is higher compared to other countries and prolonged UV exposure can be linked to developing skin cancer [58], of which Australia has the highest incidence worldwide [59, 60].

In South Australia, the Sun Smart Early Childhood Policy Guidelines state that when the UV is over 3, children need to seek shade, wear hats, protective clothing and sunscreen to play outside [61]. Parents and ECEs in this study discussed how this UV exposure risk could be mitigated when children play outdoors and described that more shaded areas are needed, which may come from trees or built shaded structures. The ECEs often identified that their centres do not have adequate shade outside and as a result could not allow children to be outdoors when the UV was high, but they described ways they mitigate this by taking children out in the morning when the UV is at its lowest compared to the middle of the day. This may require additional planning by ECEs, thus support from within the institution itself may be needed to create weather contingency plans that can be easily incorporated into the daily schedule. In addition to the institutional support, existing play spaces may require structural upgrades such as shade sales or availability of appropriate wet weather gear/protective clothing for children to use during inclement weather. In response to this, ECEs discussed that they often try to bring nature play indoors by building make-shift sand pits and colleting loose parts such as bark, leaves, sticks and rocks. Despite this, some ECEs described that bringing nature play indoors may require more cleaning and thus some ECEs were reluctant to do this.

Parents and ECE’s were asked about risky play, due to the similarities shared by nature play and risky play within the literature [62]. International members of PlaTO (play, learn, and teach outdoors) established consensus on how risky play can be defined, as a form of play that is thrilling and exciting, which involves uncertainty, unpredictability, and varying degrees of risk-taking [8]. Additionally, risky play can be characterised as children engaging in activities such climbing (trees and climbing towers), climbing up and jumping down from big rocks or small cliffs, balancing on stones or windfallen trees, shooting with bows and arrows, whittling with knives, fencing with sticks and children venturing out on their own [63, 64]. Similarly, characteristics of risky play were also described by participants when defining nature play, such as climbing trees, balancing on logs and children exploring their own capabilities. Parents and ECEs alike described risky play as being important for children; they each supported risky play and detailed how it helps children problem solve, learn their own limits and build confidence whilst giving them a sense of agency over their play. However, parents and ECEs alike described that they had safety concerns when it comes to climbing on tall structures or playing near bodies of water and there needs to be a balance between having opportunities for risky play and mitigating the risks. Similar to the finding of this study, a study by McFarland and Laird [17] found that ECEs from the USA did not encourage risky play, nor did they value it due to litigation concerns; these concerns were shared by some ECEs in this study also. Similar to the findings of Tink and colleagues [21] whom interviewed 21 Canadian educators, parents and ECEs emphasised that risky play should be age appropriate, meaning that ECEs should have an understanding about the individual child’s abilities before encouraging risky play [21]. This poses a unique challenge for ECEs, especially when staff members are casually employed and may not have enough exposure to the children to ascertain their individual abilities or have the proper training and skills necessary to assess this. It may be important for ECEs to have additional training on the perceived risk versus actual risk within their working play space, as well as what is developmentally appropriate for the age of the children.

In order for children to access and engage in nature play activities, the barriers need to be mitigated and the enablers need to be supported. One key barrier for parents and ECEs alike were that nature play activities may require more maintenance and time to pack up, creating more mess than other play activities and was identified as a key barrier. Parents and ECEs described that this barrier may be overcome by bringing a change of clothes or providing wet weather gear for the children to change into such as gum boots. Therefore, a key enabler for both parents and ECEs may be having additional facilities and equipment to help reduce clean up time; this was found to be important to educators and parents of children attending forest schools in Turkey [56]. Basbay and Atmaca [56] conducted semi-structured interviews and focus groups with 6 parents and 4 educators; they found that appropriate and sufficient equipment increased the joy and productivity of time spent in the forest, including appropriate footwear and waterproof clothes [56].

## Strengths and limitations

The strengths of this study can be attributed to the rigorous methods employed, relating to study design, sample representation and credibility. The qualitative descriptive methodology employed in the research allowed for information rich data to be collected, which allows the data to stay true to the participants point of view. By collecting data from both parents and ECEs, the phenomenon of nature play could be explored in more depth and breadth, which demonstrated credibility. Similarly, whilst there is no agreed upon or ideal sample size in qualitative research, the larger sample size of both groups in this study relative to similar studies is notable. In addition, the sample for both groups displayed a diversity of demographic characteristics, which highlights the transferability of the results across different socio-economic and South Australian contexts. Despite the strengths attached to this study there were also limitations. It is possible that using the purposive sampling method proposed that participants who had past knowledge of nature play were more likely to participate due to already having positive attitudes towards nature play. However, purposive sampling selects participants who have a story to tell, where the focus is on the phenomena of interest rather than aiming to offer a representative sample, or to draw inferences to the population (which is not the focus of qualitative research) [28]. In addition, purposive sampling allows for divergent participant perspectives [29] and we achieved this by inviting all ECEs employed at each of the study sites or parents with children attending the study sites.

## Implications for future research and practice

The findings from this study may have implications for parents, ECEs, policy makers, health, and research professionals. Based on the insights from parents and ECEs, it may be beneficial to encourage and facilitate children to engage in outdoor play spaces which have a variety of features and elements available. In particular, play spaces that have natural elements such as trees, plants, rocks, and water to afford nature play and its associated health and developmental benefits. This may be achieved by government bodies engaging with local communities by facilitating/supporting nature play events, providing resources to families about the benefits of nature play for young children or how to find nature play areas nearby, engaging with councils and early childcare facilities to implement nature play spaces locally. For children and parents to engage in nature play areas at their local early childhood setting, ECEs need to have access to these facilities, resources and support from within their institutions, government agencies and organisational leadership/management. This may involve upgrades of play spaces to include more sheltered areas to mitigate weather barriers, access to nature play resources and materials as well as supplying specialised training for professional development to direct the use of nature play spaces for child benefit. Future research may focus on developing an early childhood nature play program/curriculum for ECEs to implement within their early childhood setting. The program could include training and resources that aim to develop ECE knowledge about the benefits of nature play, creating resources for parents, nature play pedagogical skill workshops and management of nature play resources/spaces. Additionally, future research may benefit from implementing, and evaluating the effectiveness of the program within early childhood settings, in terms of measuring ECE/parent knowledge about nature play and child health outcomes.

## Conclusion

Exploring the perspectives of nature play for parents and ECEs within a South Australian context has provided important insights into the barriers and enablers of nature play engagement for young children. Adults may be considered gatekeepers to play and inherently restrict or facilitate children’s access to nature play spaces. There is a plethora of factors that impact these choices for parents and ECEs such as time, resourcing, weather, proximity, completing child interests and concerns around safety. These factors may be influenced by parent and ECEs personal experiences with nature play, knowledge about nature play and positive affirmations about nature play. Parents and ECEs may be more inclined to facilitate nature play engagement if they are provided with nature play resources and guidance on how to use those resources. ECE’s may need additional institutional support to ensure consistent training and knowledge about nature play is delivered.

## Supporting information

S1 Appendix COREQ checklist. (ZIP)

S2 Appendix Interview guide. (ZIP)
