# Supplementary figures and images for: Parent and early childhood educator perspectives of unstructured nature play for young children: A qualitative descriptive study
Source: PLoS One. 2023 Jun 7;18(6):e0286468. doi: 10.1371/journal.pone.0286468 (PMC10246796; doi:10.1371/journal.pone.0286468)

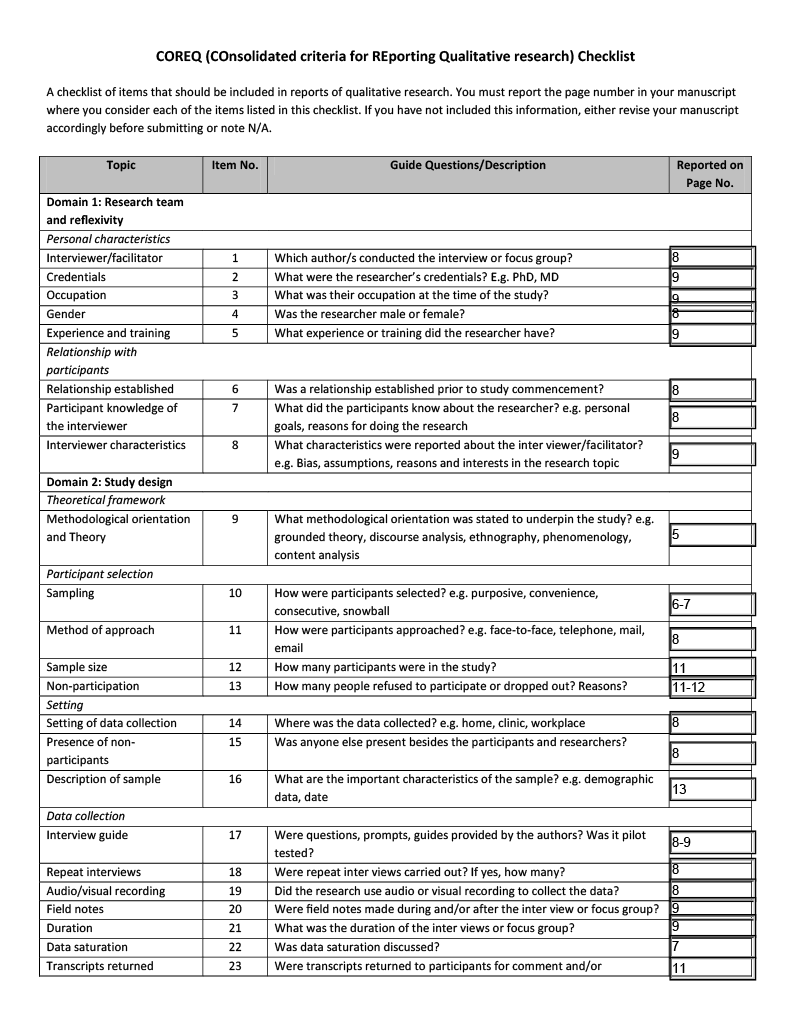

Supplement: S1 Appendix — (ZIP) [file pone.0286468.s001.zip › S1 Appendix. COREQ Checklist_Page 1 of 2.tiff]

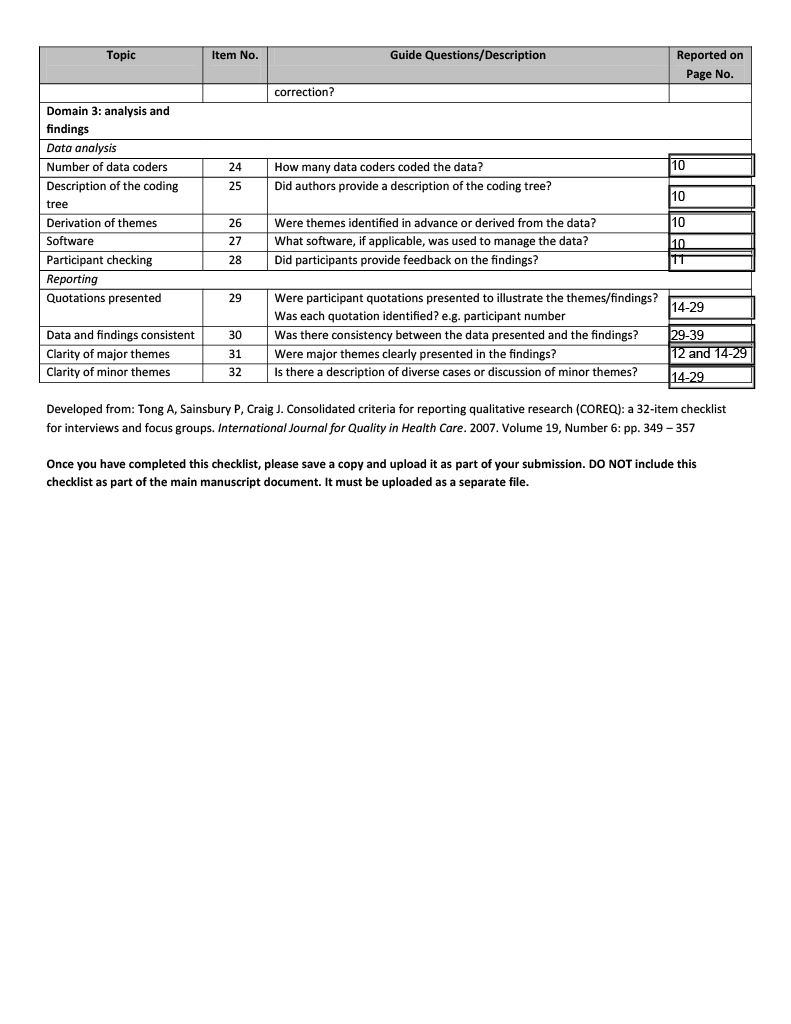

Supplement: S1 Appendix — (ZIP) [file pone.0286468.s001.zip › S1 Appendix. COREQ Checklist_Page 2 of 2.tiff]

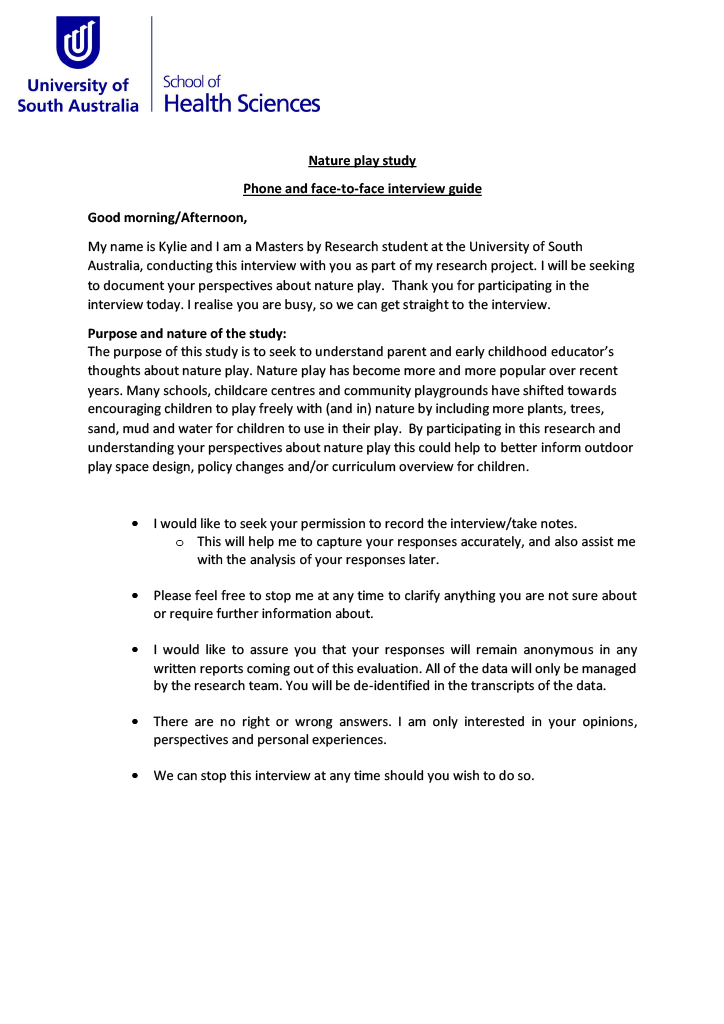

Supplement: S2 Appendix — (ZIP) [file pone.0286468.s002.zip › S2 Appendix. Interview Guide Page 1 of 3.tiff]

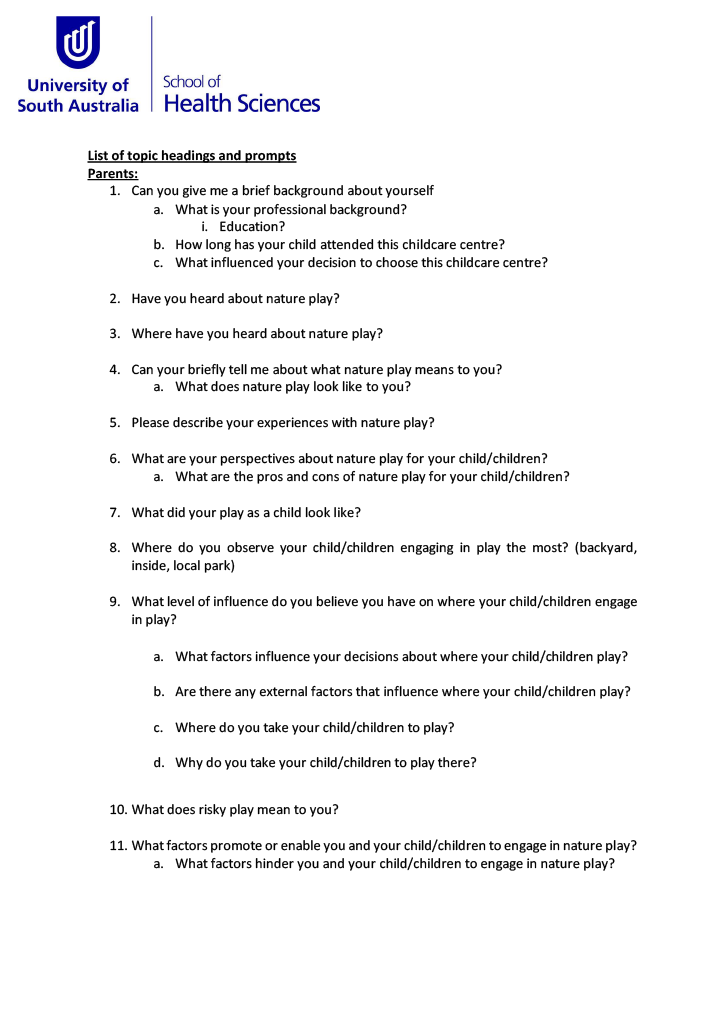

Supplement: S2 Appendix — (ZIP) [file pone.0286468.s002.zip › S2 Appendix. Interview Guide Page 2 of 3.tiff]

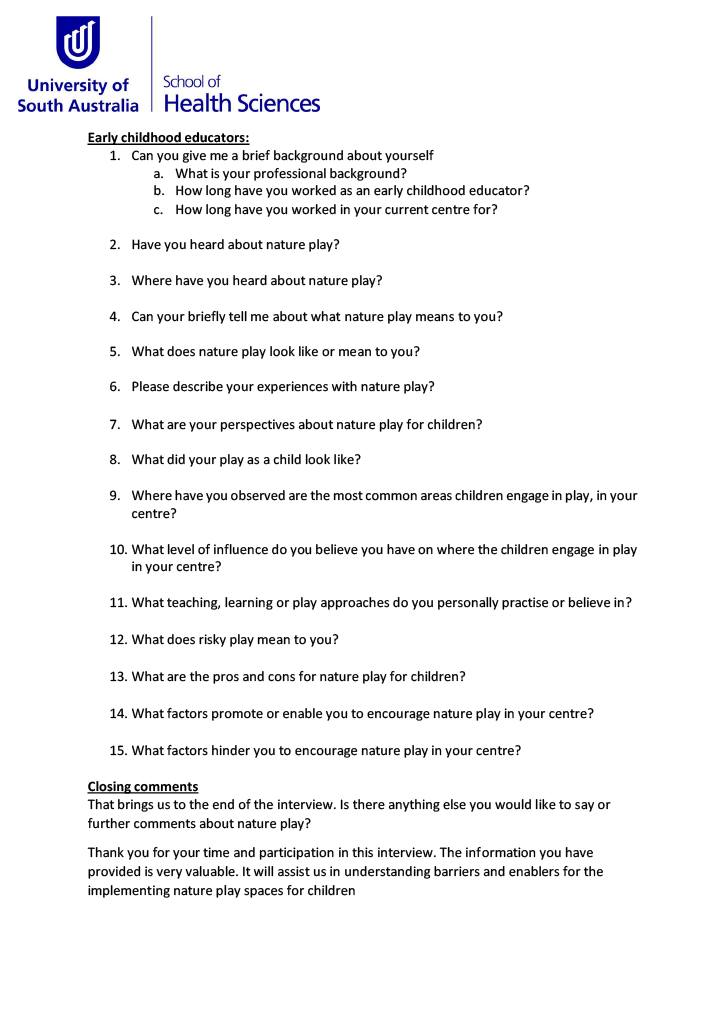

Supplement: S2 Appendix — (ZIP) [file pone.0286468.s002.zip › S2 Appendix. Interview Guide Page 3 of 3.tiff]
